# Combination therapies for primary hepatic neuroendocrine carcinoma: a case report

**DOI:** 10.1186/s40792-017-0378-z

**Published:** 2017-09-11

**Authors:** Richi Nakatake, Morihiko Ishizaki, Kosuke Matui, Hiroaki Yanagimoto, Kentaro Inoue, Masaki Kaibori, Yusai Kawaguchi, Masanori Kon

**Affiliations:** Department of Surgery, Kansai Medical University, 2-5-1 Shinmachi, Hirakata, Osaka 573-1010 Japan

**Keywords:** Primary hepatic neuroendocrine carcinoma, Liver resection, Hepatectomy

## Abstract

**Background:**

Primary hepatic neuroendocrine carcinomas are extremely rare. Because of the rarity of PHNEC, its clinical features and treatment outcomes are not well understood. A proper diagnosis and the correct therapeutic approach therefore remain clinically challenging.

**Case presentation:**

A 67-year-old man was admitted to our department because of a liver tumor. Computed tomography revealed a single liver tumor 50 mm in diameter and located in the S3 region. Biopsy and imaging findings resulted in a diagnosis of primary hepatic neuroendocrine carcinoma. Left lateral segmentectomy was performed. Immunohistochemically, the tumor cells were positive for synaptophysin, chromogranin A, and CD56. Ki-67 was positive in > 90% of the tumor cells. The final diagnosis was primary hepatic neuroendocrine carcinoma. The patient suffered two episodes of lymph node recurrence. Nonetheless, the tumor was excised to prolong survival. Thus, after lymphadenectomy, he received adjuvant chemotherapy for 6 months. Two years after surgery, the patient remains alive and in good general condition.

**Conclusions:**

In most cases, primary hepatic neuroendocrine carcinoma, while extremely rare, has a poor prognosis. At present, surgical resection is a priority for curative treatment, but in patients with recurrence, combined therapies are recommended.

## Background

Primary hepatic neuroendocrine carcinomas (PHNECs) are extremely rare, with roughly 64 cases reported in the English-language literature until 2016 [1–26]. Because of the rarity of PHNEC, its clinical features and treatment outcomes are not well understood. A proper diagnosis and the correct therapeutic approach therefore remain clinically challenging. We herein report a case of PHNEC.

## Case presentation

**Fig. 1:**
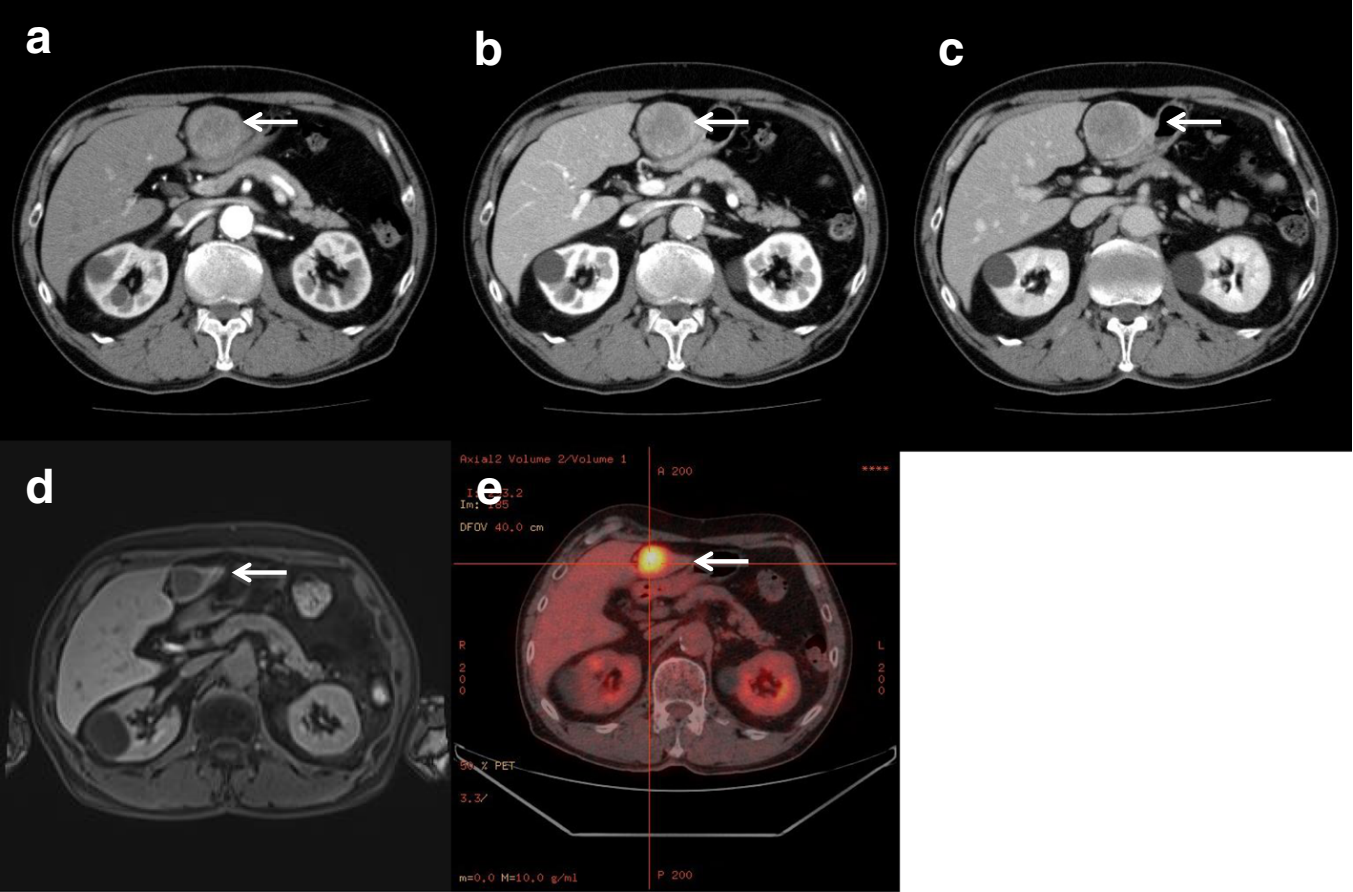
**a**–**c** Liver dynamic computed tomography (CT). **a** Arterial phase; **b** Portal phase; **c** Delayed phase. The tumor shows low signal intensity, with an unclear border in each phase (arrows). **d** Gd-EOB-DTPA-MRI shows tumor nodules in the liver and a low-intensity and hypo-vascular tumor in segment 3 (arrow). **e** Positron emission tomography-CT. The SUV max of the tumor in S3 of the liver is 10 (arrow)

A 67-year-old male was admitted to our hospital for evaluation and management of a symptomatic liver mass. His past medical history included bladder cancer for a postoperative follow-up. Liver dynamic computed tomography (CT) showed a low-density mass in the S3 area (Fig. 1a–c) and magnetic resonance imaging (MRI) a mass with different signal patterns (Fig. 1d). On positron emission tomography (PET)-CT, the SUV max of the tumor in S3 of the liver was 10 (Fig. 1e). Both MRI and PET-CT confirmed a single liver tumor 50 mm in diameter located in the S3 region. Because of the patient’s past medical history, liver metastasis of bladder cancer or other cancers was suspected. Thus, we performed the liver biopsy preoperatively. The histological reports of biopsy revealed a solitary epithelial neoplasm. Immunohistochemistry demonstrated the expression of synaptophysin and CD56 (not chromogranin A). The patient was diagnosed with primary neuroendocrine carcinoma based on the biopsy results and imaging findings. To identify the primary neoplasm, chest and abdominal CT, upper and lower endoscopy, and abdominal MRI were performed. None of these examinations revealed a primary lesion outside the liver.

Upon presentation, the patient was afebrile, had no history of weight loss, and his appetite was good. His laboratory test results did not reveal any evidence of liver dysfunction. Antibodies against hepatitis B virus and hepatitis C virus surface antigens were negative. Serum tumor markers alpha-fetoprotein, carcinoembryonic antigen, and cancer antigen 19–9 were within the normal range, but neuron-specific enolase levels increased (27.3 mg/dl). The patient was therefore diagnosed with primary liver cancer and a resection of the lateral segment of the liver was planned. After left lateral segmentectomy, the postoperative course was uneventful and the patient was discharged on the tenth postoperative day.

**Fig. 2:**
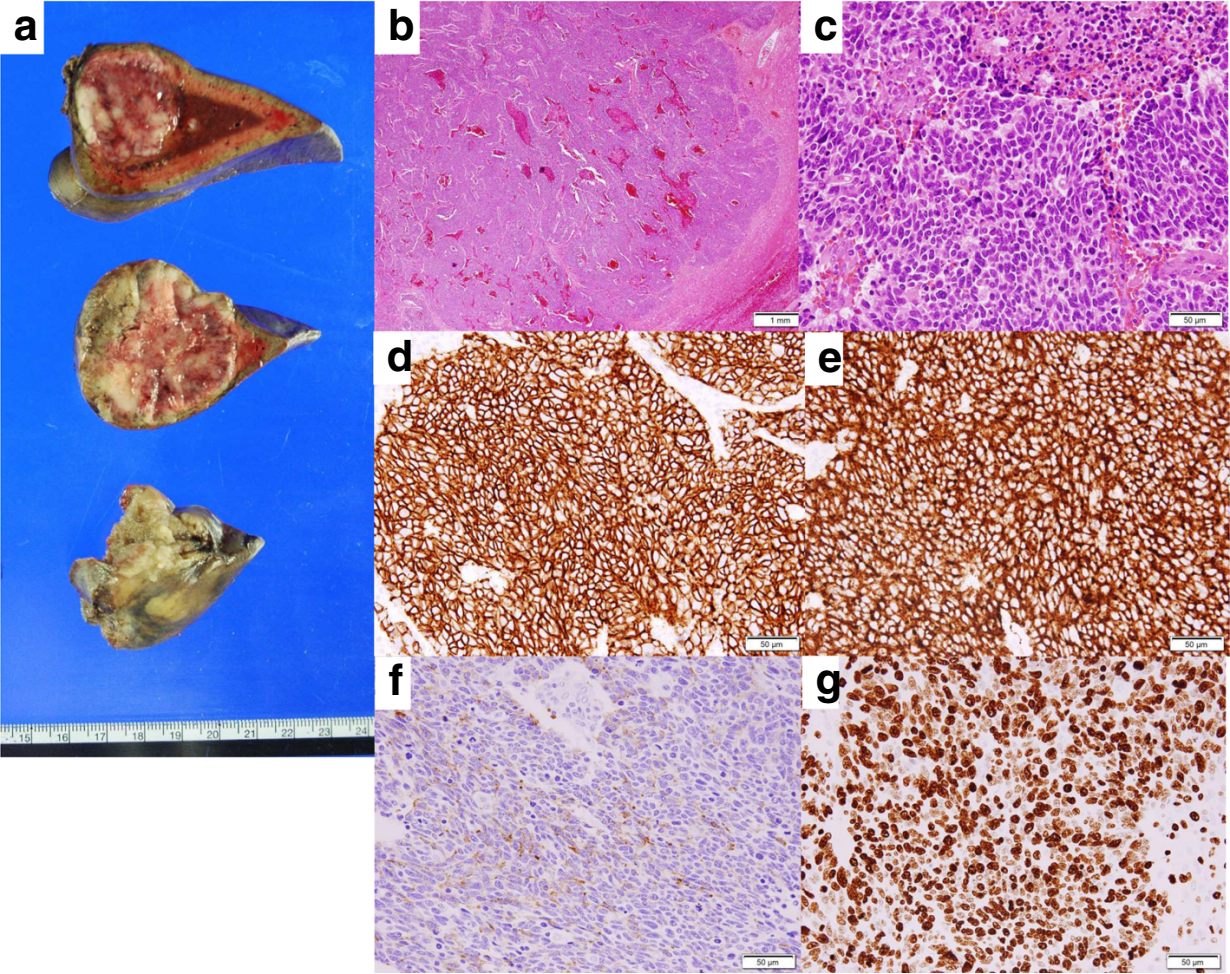
Macroscopic and histopathological findings of the resected specimen. **a** The lateral segment consists of a solid mass. **b**, **c** Hematoxylin-eosin staining shows the enlarged nuclei and condensed chromatin of the tumor cells. **d**–**g** Immunopathological examination. The tumor cells are positive for CD56 (d), synaptophysin (**e**), and chromogranin A (**f**). Ki-67 was positive in > 90% of the tumor cells (**g**)

The pathology report revealed a solitary epithelial neoplasm with a well-developed vascular network (Fig. 2b, c) and no tumor invasion of vein and bile duct. The sub-classification of NEC categorized into small type. The morphology of this tumor was homogeneous. There existed no components of adenocarcinoma and well-differentiated NET. Immunohistochemistry demonstrated expression of synaptophysin, chromogranin A, and CD56. Ki-67 was positive in > 90% of the tumor cells (Fig. 2d–g). The diagnosis of a primary neuroendocrine carcinoma of the liver was established (PHNEC grade 3). Resection margins were tumor-free (R0 resection).

**Fig. 3:**
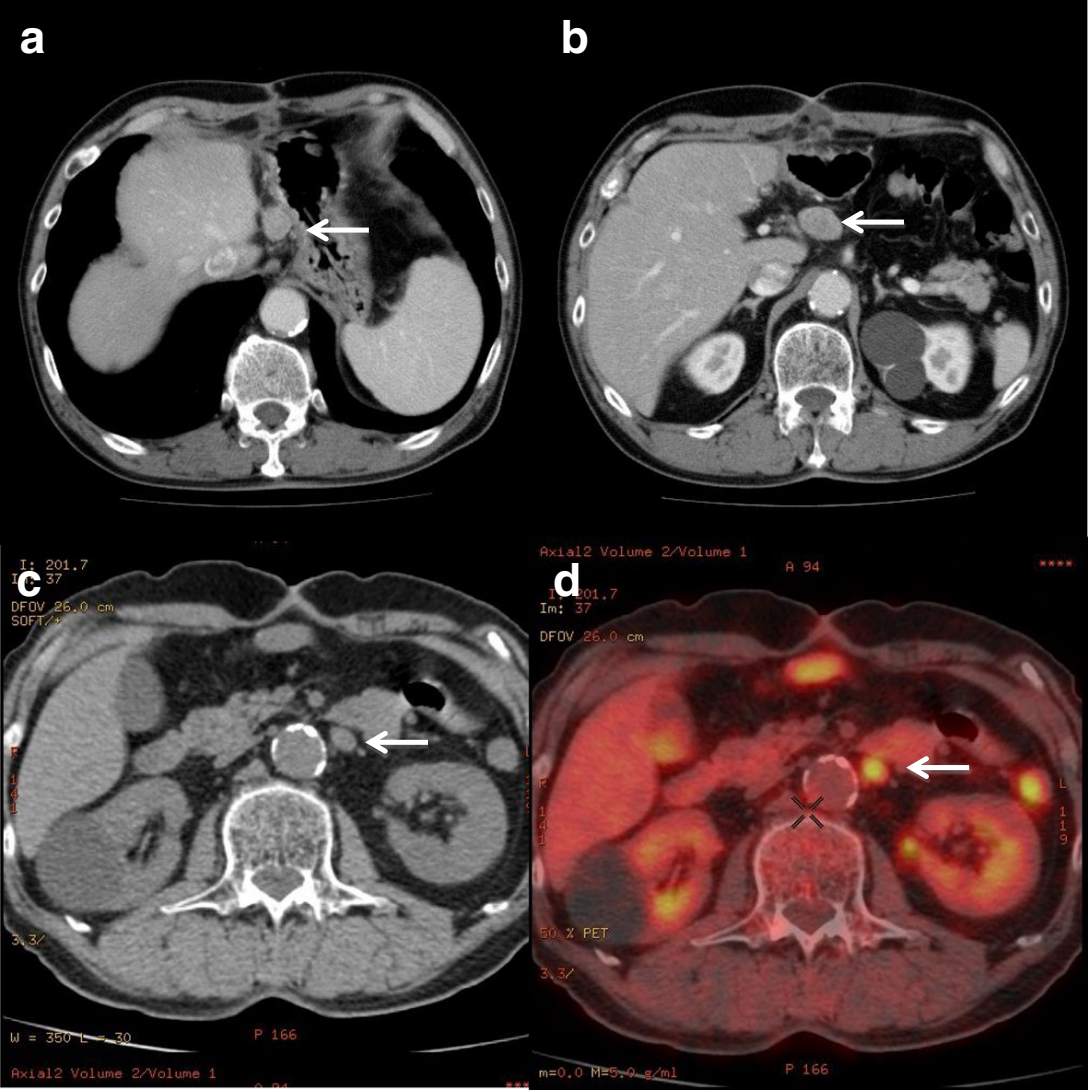
**a**, **b** Computed tomography (CT) shows lymph node recurrence (a; #3, b; #8, arrows) . **c**, **d** CT and positron emission tomography-CT. **c** CT shows recurrence (arrow) in the left renal vein lymph nodes. **d** The SUV max of the tumor was 4.6 (arrow)

Three months after surgery, the patient suffered lymph node (#3, 8) recurrence (Fig. 3a, b) and underwent lymphadenectomy. Four months after the second surgery, recurrence was detected in the left renal vein lymph nodes (Fig. 3c, d). The tumor burden could be reduced using an antitumor agent (cisplatin + irinotecan) for 4 months to allow subsequent excision of the tumor. Thus, after lymphadenectomy, he received adjuvant chemotherapy (cisplatin + irinotecan) for 6 months. Two years after the first surgery, the patient remains in good general condition.

### Discussion

In the fourth edition of the WHO Classification of Tumors of the Digestive System, published in 2010, the term “neuroendocrine neoplasms” replaced the previously used “neuroendocrine tumors” [27]. Neuroendocrine neoplasms can be categorized into three grade-based groups. Low- and intermediate-grade neuroendocrine neoplasms are neuroendocrine tumors grades 1 and 2, respectively; high-grade neuroendocrine neoplasms are neuroendocrine carcinomas. Neuroendocrine tumors develop in organs or tissues that contain peptide and amine-producing cells and exhibit different hormonal profiles depending on their site of origin [28, 29]. Overall, approximately 57.0 and 27.0% of all neuroendocrine tumors arise within the gastroenteropancreatic and bronchopulmonary systems, respectively [30]. Within the gastrointestinal tract, most neuroendocrine tumors occur in the rectum (17.2%), jejunum/ileum (13.4%), and pancreas (6.4%) [30].

Neuroendocrine tumors are diagnosed by pathologic confirmation. On hematoxylin and eosin staining, the tumors may demonstrate an insular, trabecular, or glandular cell arrangement [7]. Immunohistochemical staining of these tumors reveals immunoreactivity to specific markers, including chromogranin A, neuron-specific enolase, and synaptophysin [31, 32]. The exclusion of an extrahepatic origin of the tumors and a pathological analysis of the neuroendocrine carcinoma are needed for the diagnosis of PHNEC. Because the liver is the most frequent metastatic site of neuroendocrine carcinoma, PHNEC must also be diagnostically differentiated from metastatic hepatic neuroendocrine carcinoma [14].

There is no standard for the therapy of PHNEC. Currently, surgery is the only curative option and provides the most favorable outcome, including long-term survival [15]. Only 33 reports of surgery in PHNEC patients have been published in the literature (Table 1) [2, 4, 7, 10, 11, 13–15, 19, 20, 22–24, 26]. Park et al. reported on three patients with resectable tumors who were alive 17.7 months after treatment (range, 15.2–36.9 months) and on nine patients whose tumors could not be surgically removed but who survived for 11.3 months (range, 0.7–41.7 months) [20]. Even in a patient with a giant tumor, curative resection allowed long-term survival [23]. The preferred treatment for PHNEC for tumors without distant or lymph node metastasis is surgical resection [19]. Surgical resection for PHNEC is an independent predictor of survival. However, surgery alone is rarely curative, since the vast majority in patients with PHNEC undergoing resection will develop recurrences. It is a reason why adjuvant chemotherapy after curative resection should be considered, although no prospective studies are available to support this practice. While resection of all tumors could lead to a higher survival rate and better outcomes, many patients will still require combined therapy, such as transcatheter arterial chemoembolization, chemotherapy, and radiofrequency ablation [26].

**Table 1:** Summary of reported surgical cases of neuroendocrine carcinoma

First author (year)	ref	Age/sex	Symptom	Treatment	Tumor size	Details of	Recurrence	Details of	Treatment after	Outcome	Survival
				before surgery	(cm)	Metastasis	(months)	recurrence	recurrence		(months)
Hsueh (1983)	[2]	8/F	Dizziness, fatigue	Chemotherapy	17	LN, lung	None	None	None	Died	4
Zanconati (1996) [4]	4	56/M	Abdominal discomfort,Jaundice	None	5	ND	3	Liver	None	Died	5
Pilichowska (1999) [7]	7	57/M	Hypochondria mass	None	8.2	Lung	ND	Liver	None	Died	16
Ishida (2003) [10]	10	72/M	None	None	3	LN	ND	ND	None	ND	ND
Garcia (2006) [11]	11	50/M	None	None	5	None	4	Liver, mesenteric	TACE and Chemotherapy	Died	16
Yang (2009) [13]	13	65/M	Epigastric pain	None	7.5	LN	3	Liver	None	Died	12
		56/F	None	None	ND	None	None	None	ND	Alive	36.9
		68//F	Fatigue	None	ND	None	None	None	ND	Alive	18
		51/F	None	Chemotherapy	ND	None	6.2	ND	ND	Alive	15.2
Akahoshi (2010) [14]	14	64/M	None	None	1.5	None	None	None	ND	Alive	3
Huang (2010) [15]	15	51/M	Abdominal pain	TACE	ND(Multiple)	None	48	Liver	ND	Alive	107
		34/M	Medical exam	TACE	ND(Multiple)	None	None	None	ND	Alive	98
		52/F	Diarrhea	None	ND(Multiple)	None	5	liver	ND	Alive	47
		59/M	Medical exam	None	ND	None	None	None	None	Alive	34
		54/M	Medical exam	None	ND	None	None	None	None	Alive	24
		43/M	Abdominal pain	None	ND(Multiple)	None	None	None	None	Alive	15
		50/F	Medical exam	None	ND(Multiple)	None	5	Liver	ND	Alive	14
		37/M	Diarrhea	None	ND(Multiple)	None	1	Liver	PEIT	Alive	13
		58/F	Medical exam	None	ND	None	39	ND	ND	Alive	148
		56/F	Medical exam	None	ND(Multiple)	None	5	Liver	ND	Alive	33
		50/M	Medical exam	None	ND(Multiple)	None	3	Liver	ND	Alive	12
Shinkawa (2013) [19]	19	73/M	Medical exam	None	5	None	4	Bone,LN	ND	Died	6
Kim (2013) [21]	20	67/F	nausea	None	9	LN	None	None	None	Alive	3
Kano (2014) [22]	22	73/M	Medical exam	None	3	LN	6	Liver	Chemotherapy	Alive	10
Sotiropoulos (2014) [23]	23	19/F	Symptomatic liver mass	None	27	None	None	None	None	Alive	24
Aboelenen (2014) [24]	24	51/M	Abdominal pain	None	20	None	None	None	None	Alive	6
Wang (2014) * [26]	26										

*ND* not discribed, *LN* lymph node, *TACE* transcatheter arterial chemoembolization, *PEIT* percutaneous ethanol injection therapy

*There are 8 surgical cases, but personal information are not described

There is still no report of typical treatment for recurrence in PHNEC. In Wang’s series, combined therapy resulted in better outcomes than monotherapy even in patients with recurrence [26]. Tumor progression can be controllable with antitumor agents, and tumor burden can be reduced without the evidence of other recurrence, allowing subsequent excision in the tumor. Further studies are needed to more accurately determine the clinical features of PHNEC.

## Conclusions

In conclusion, PHNEC is a rare liver primary tumor with unique specificity regarding its occurrence and development. At present, surgical resection is a priority for curative treatment, but in patients with recurrence, combined therapies are recommended.

## Abbreviations

CT: Computed tomography
MRI: Magnetic resonance imaging
PHNEC: Primary hepatic neuroendocrine carcinoma
